# *Staphylococcus argenteus* bacteremia in the Republic of Korea

**DOI:** 10.1128/spectrum.02798-23

**Published:** 2024-01-10

**Authors:** Minkyeong Lee, Yunsang Choi, Seong Jin Choi, Song Mi Moon, Eu Suk Kim, Hong Bin Kim, Soyeon Ahn, Hyunju Lee, Jaeeun Kim, Dong Woo Shin, Jinki Yeom, Jeong Su Park, Kyoung-Ho Song

**Affiliations:** 1 Department of Internal Medicine, Seoul National University Bundang Hospital, Seoul National University College of Medicine, Seongnam, Republic of Korea; 2 Department of Medical Research Collaborating Center, Seoul National University Bundang Hospital, Seongnam, Republic of Korea; 3 Department of Pediatrics, Seoul National University Bundang Hospital, Seoul National University College of Medicine, Seongnam, Republic of Korea; 4 Department of Biomedical Science, College of Medicine, Seoul National University, Seoul, Republic of Korea; 5 Department of Laboratory Medicine, Seoul National University Bundang Hospital, Seoul National University College of Medicine, Seongnam, Republic of Korea; 6 Department of Microbiology and Immunology, College of Medicine, Seoul National University, Seoul, Republic of Korea; Icahn School of Medicine at Mount Sinai, New York, New York, USA

**Keywords:** *Staphylococcus aureus*, *Staphylococcus argenteus*, bacteremia, MALDI-TOF MS, Republic of Korea

## Abstract

In 2015, *Staphylococcus argenteus* and *Staphylococcus schweitzeri* were proposed as new species, distinct from *Staphylococcus aureus* and collectively referred to as the *S. aureus* complex. However, no clinical reports of these new species exist in Korea. Upon the application of matrix-assisted laser desorption/ionization time-of-flight mass spectrometry (MALDI-TOF MS) for all bloodstream isolates since September 2022, *S. argenteus* was identified in one patient. Therefore, we aimed to search for new species among the archives of the *S. aureus* bacteremia cohort and describe their clinical and microbiological characteristics. Among the 691 archived *S. aureus* isolates between 2012 and 2018, one was identified as *S. argenteus* via MALDI-TOF MS. Both *S. argenteus* isolates (one in 2022) were obtained from patients with extensive pneumonia accompanied by bacteremia and both cases had fatal outcomes. They harbored multiple virulence genes (*clfA, clfB, fnbpA, sdrC, sdrD, sdrE, bbp, cna, see, seg, sei, blaZ, fnbpB,* and *map*) but did not harbor *mecA* and *pvl*. No matched sequence type (ST) was found in either isolate, and both *S. argenteus* isolates were closely related to ST1594, ST1593, ST1793, and ST1303, which belonged to *S. argenteus. S. argenteus* accounted for <1% of the *S. aureus* complex but had clinical characteristics similar to *S. aureus*. Therefore, clinicians should be aware of these factors to avoid misidentifying these strains as coagulase-negative staphylococci, and appropriate reporting is required to minimize confusion.

**IMPORTANCE:**

*Staphylococcus argenteus*, a member of *Staphylococcus aureus* complex, has been reported as an important pathogen that causes clinically invasive infections in humans similar to *S. aureus*. Clinical isolates of *S. argenteus* have been reported across the world, showing a large geographical difference in prevalence and genomic profile. However, there have been no clinical reports regarding this new species in Korea. This is the first report to investigate the clinical and genetic characteristics of *S. argenteus* identified in patients with bacteremia, and the proportion of *S. argenteus* bacteremia among *S. aureus* bacteremia cohort in Korea.

## OBSERVATION

*Staphylococcus aureus* is a clinically important pathogen that causes various infections ranging from skin and soft tissue infections to infective endocarditis (1). In 2015, *Staphylococcus argenteus* and *Staphylococcus schweitzeri* were identified as new species, distinct from *S. aureus* (2). These new species are indistinguishable from *S. aureus* using conventional routine diagnostics, such as microscopy, colony morphology, and coagulase assays and are collectively referred to as the *S. aureus* complex. However, they can be distinguished via peptidoglycan composition analysis, molecular typing, such as matrix-assisted laser desorption/ionization time-of-flight mass spectrometry (MALDI-TOF MS), genotyping, including multi-locus sequence typing (MLST), and whole-genome sequencing (3). Since new diagnostic methods based on molecular typing or genotyping have been introduced and are widely used, clinicians may encounter *S. argenteus* more frequently based on clinical microbiology laboratory results. However, no clinical reports on these new species exist in Korea; thus, they remain largely unrecognized. Therefore, we aimed to investigate the presence of these species in an archived *S. aureus* bacteremia cohort and describe their clinical and microbiological characteristics.

Since September 2022, MALDI-TOF MS has been applied to all positive blood cultures of patients with bacteremia at the study hospital. *S. argenteus* was identified in the blood culture of one patient (strain 1) on 4 December 2022. Stored isolates from the *S. aureus* bacteremia cohort were re-evaluated using MALDI-TOF MS to identify possible misidentifications. This study involved the *S. aureus* bacteremia cohort treated between May 2012 and December 2018. Four cohorts from which data were prospectively collected over the study period were included, and their detailed information is described in the Acknowledgments section (4–7).

Isolates were evaluated using a MALDI Biotyper Sirius (Bruker Daltonics GmbH & Co. KG, Bremen, Germany) with the MBT compass reference library (version 11.0) containing *S. argenteus* and *S. schweitzeri* in the strain list, which was highly sensitive and specific for distinguishing *S. argenteus* from *S. aureus* (8). MLST was performed and the sequence type (ST) was analyzed based on the MLST scheme for *S. aureus* using the *S. aureus* pubMLST database (https://pubmlst.org/organisms/staphylococcus-aureus) (9). Phylogenetic analysis was performed by aligning concatenated MLST data using CLUSTALW and constructing the phylogenetic tree using the neighbor-joining method in MEGA version 11. *S. argenteus* isolates used in this study were distinguished from the reference collection based on phylogenetic clustering. Furthermore, 73 STs were downloaded from the MLST database and selected as reference sequences based on the initial analysis of each *S. aureus* MLST locus using the same database (not shown).

Genomic DNA was extracted using an E.Z.N.A. Stool DNA Kit (Omega Bio-tek, USA) according to the manufacturer’s protocol. Library preparation from isolated DNA and *de novo* whole-genome sequencing (WGS) were performed by Macrogen (Seoul, Republic of Korea). High-quality DNA was used for constructing the library with the help of the TruSeq Nano DNA kit. The WGS was done on the Illumina sequencing by synthesis platform (Illumina Inc., San Diego, CA, USA).

The antimicrobial susceptibility of *S. argenteus* isolates was determined using the Sensititre Gram-positive GPALL1F AST Plate (Thermo Fisher Scientific, MA, USA), a commercially available broth microdilution method-based kit. The test was performed manually according to the manufacturer’s instructions, and the results were interpreted based on the Clinical and Laboratory Standards Institute breakpoints for *S. aureus* (10). The isolates were investigated for the presence of genes encoding *mecA, blaZ,* Panton-Valentine leukocidin, adhesins, enterotoxins, toxic shock syndrome toxin, and exfoliative toxins using primers as described in previous reports (11–13). The 95% confidence interval of the proportion of *S. argenteus* among *S. aureus* complexes was estimated using the binomial exact method.

Among the 691 isolates of *S. aureus* bacteremia cohort from the study hospital, one was identified as *S. argenteus* (strain 2). The estimated proportion of *S. argenteus* among *S. aureus* complex was 0.14% (95% confidence interval 0%–0.8%). The clinical and microbiological characteristics of both *S. argenteus* isolates (strains 1 and 2) are presented in Table 1. Both isolates were identified from the blood cultures of patients with extensive pneumonia accompanied by bacteremia. Both patients died from multi-organ failure despite intensive care.

**TABLE 1:** Clinical and microbiological characteristics of two cases of *Staphylococcus argenteus* bacteremia^*b*^

Clinical characteristics	Case 1 (2022)	Case 2 (2017)
Age	70	81
Sex	Male	Male
Relevant comorbidities	Video-assisted pulmonary lobectomy, chemotherapy, neutropenia (ANC 980 cells/mm^3^)	Chronic obstructive pulmonary disease
Symptom		
Onset	5 days before admission	1 day before admission
Fever	+	+
Dyspnea	+	+
Cough	+	−
Infection type	Healthcare-associated pneumonia	Community-acquired pneumonia
Clinical sample	Blood	Blood
Date of sample collection	04.12.2022	20.02.2017
Treatment		
Empirical antibiotics	Ceftizoxime, amikacin	Piperacillin/tazobactam
Definitive antibiotics	Cefazolin	Nafcillin
Mechanical ventilation	+	+
Continuous renal replacement therapy	−	+
Outcome	Deceased	Deceased
Microbiological characteristics		
Multi-locus sequence typing (allelic profile)		
*arcC*	151	151
*aroE*	755	47
*glpF*	20	8
*gmk*	101	34
*Pta*	145	175
*Tpi*	150	180
*yqiL*	131	169
Sequence type	8342	8343
Antimicrobial susceptibility test (MIC, mg/L)^*a*^		
Oxacillin	0.5 (S)	≤0.25 (S)
Rifampin	≤0.5 (S)	≤0.5 (S)
Clindamycin	≤0.5 (S)	≤0.5 (S)
Vancomycin	1 (S)	2 (S)
Linezolid	4 (S)	4 (S)
Levofloxacin	>4 (R)	≤0.25 (S)
Trimethoprim-sulfamethoxazole	≤0.5/9.5 (S)	≤0.5/9.5 (S)

^*a*^ Antimicrobial susceptibility test was performed using the Sensititre Gram-positive GPALL1F AST Plate (Thermo Fisher Scientific, MA, USA), a commercially available broth microdilution method-based kit, and interpreted according to Clinical and Laboratory Standards Institute breakpoints for *Staphylococcus aureus.*

^*b*^ ANC, absolute neutrophil count and MIC, minimal inhibitory concentration.

**Fig 1:**
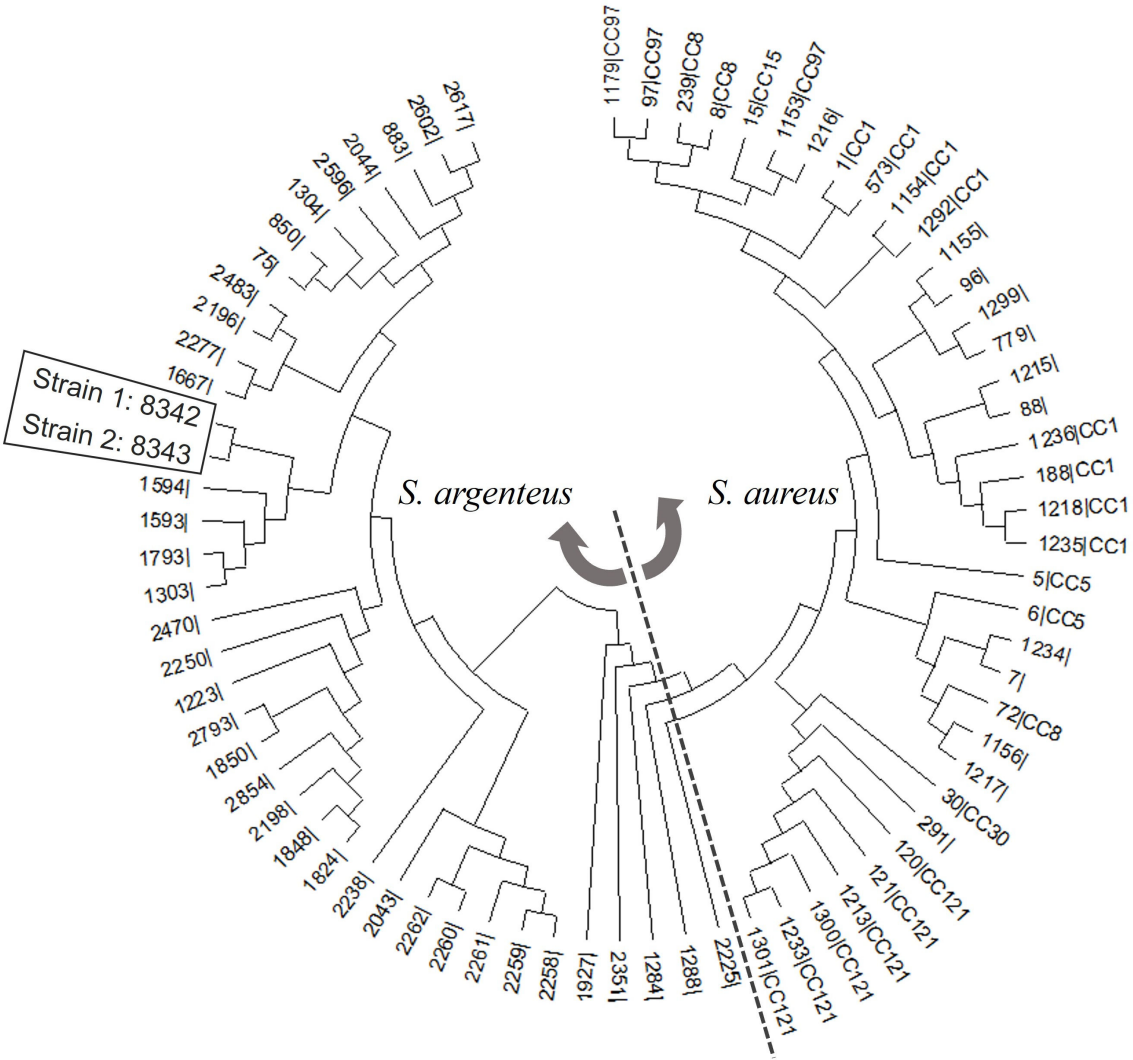
Phylogenetic tree of two *S*. *argenteus* isolates and 73 STs downloaded from the MLST website (http://www.mlst.net). The tree was constructed based on concatenated sequences of seven MLST loci using the neighbor-joining method.

No ST was consistent with both isolates using the *S. aureus* pubMLST database on 17 April 2023. Therefore, the allelic profiles of both isolates were deposited in the MLST database, and the new STs were classified as 8342 and 8343. Phylogenetic analysis revealed that strains 1 and 2 were closely related to ST1594, ST1593, ST1793, and ST1303, which belonged to *S. argenteus* (Fig. 1). Both isolates were susceptible to oxacillin, rifampin, and vancomycin (Table 1). Strain 1 was resistant to levofloxacin. Both isolates harbored genes associated with virulence (*clfA, clfB, fnbpA, sdrC, sdrD, sdrE, bbp, cna, see, seg,* and *sei*). Additionally, *blaZ, fnbpB,* and *map* existed only in strain 1. Neither isolate had *mecA* and *pvl*.

This is the first report to investigate the clinical and microbiological characteristics of *S. argenteus* identified in patients with bacteremia and the proportion of *S. argenteus* bacteremia among *S. aureus* bacteremia in Korea. *S. argenteus* has been reported worldwide, particularly high in Australia, Thailand, and Taiwan accounting for 11.9%–25% of *S. aureus*-associated infections (14–16). However, <1% have been reported in Europe and East Asia, including the Netherlands, Belgium, Sweden, Denmark, China, and Japan, revealing a large geographical difference (17–22). The proportion of *S. argenteus* among *S. aureus* complex in this study was consistent with that of reports from neighboring countries, China and Japan (20, 21). The estimated proportion of *S. argenteus* in this study was low; nonetheless, the incidence of *S. argenteus* infection may change as global interaction/immigration increases and requires further monitoring.

Both *S. argenteus* isolates were associated with community- and healthcare-acquired pneumonia resulting in fatal outcomes, consistent with previously reported high virulence (15, 16). Strains 1 and 2 were classified as new ST8342 and ST8343, respectively. The most common ST in *S. argenteus* was ST2250, followed by ST1123 (23). As ST composition indicates geographical diversity, further studies are needed to determine whether the *S. argenteus* ST in Korea differs from those of other regions (23). Various virulence genes encoding adhesins, enterotoxins, toxic shock syndrome toxin, and exfoliative toxin were identified in this study, consistent with previous reports that *S. argenteus* and *S. aureus* share a significant proportion of genes encoding virulence factors (3, 24). Both isolates were methicillin-susceptible*,* as *S. argenteus* is associated with lower antimicrobial resistance than *S. aureus* (16, 17). However, penicillin-resistant *S. argenteus* is relatively common and carries *blaZ*, similar to strain 1 (23).

Although, *S. argenteus* accounted for <1% of the *S. aureus* complex in Korea, it was highly virulent and associated with severe infections as *S. aureus*. However, *S. argenteus* may be misrecognized as coagulase-negative staphylococci in clinical settings because it is an unfamiliar species, especially in regions with a low incidence, such as Korea. Therefore, we believe reporting *S. argenteus* as a member of the *S. aureus* complex is necessary to avoid confusion with coagulase-negative staphylococci, as suggested by the ESCMID Study Group for Staphylococci and Staphylococcal Diseases in 2019 (3).

## Data Availability

The complete genome sequences of the two *S. argenteus* strains have been deposited in GenBank under accession numbers SAMN38109731 (SNUBHSAr1) and SAMN38109732 (SNUBHSAr2).

## AFTER EPUB

*[This article was published on 10 January 2024 with an error in Acknowledgments. The error was corrected in the current version, posted on 16 January 2024.]*
